# Dissertation on Tooth-Ache

**Published:** 1845-12

**Authors:** John Harris


### ARTICLE II.

*Dissertation on Tooth-Ache.* Delivered before the American Society of Dental Surgeons, at their Sixth Annual Meeting, held in the City of New York, August, 1845. By JOHN HARRIS, M. D., D. D. S.

***Mr. President, and Gentlemen***
***of the American Society of Dental Surgeons:***

BEFORE entering upon the discharge of the duty which you assigned me at your last meeting, I would embrace the opportunity to express my most grateful appreciation of the honor which you conferred upon me, in making me a member of your respected and valuable body, and in appointing me to address you on the present occasion, on some subject connected with dental theory or practice. I regret, that, until the present time, circumstances beyond my control, have prevented me from attending any of your annual convocations, and from sharing in your sacrifices and toils, and manifesting that zeal which the cause of science, our profession, and humanity demand. The members of this association have already contributed largely to the advancement of the science and art of dental surgery ; they have also added to the respectability of the pursuit, and judging from what has been achieved by comparatively so few, and in so short a period, I am encouraged to believe that the day is not distant when the practice of this branch of the curative art will be as much respected as is that of general medicine and surgery.

I need not speak of the many difficulties which you have had to encounter, nor of the sacrifice of time and money which many of you have had to make in attending the meetings of the society. These have all, all, been disregarded in the noble objects contemplated by this association. Many of those, who at first, regarded its organization with a jealous eye, have subsequently sought, and some have obtained admission to membership in it, and thus the number of its members have been yearly gradually increasing.

The period of the existence of this society may well be regarded as constituting the most interesting and important era in the history of this branch of medicine. Such has been the great interest manifested by its members, that its meetings have been numerously attended. Every part of our widely extended country, has, from year to year, been represented by some of our ablest and best practitioners, bringing with them the results of their professional researches, for the mutual benefit of all. The advantages that have resulted from this sort of intercommunication have been great, and but for which, many valuable discoveries and improvements might still have remained in the possession of the few by whom they were made.

But without further introduction, I shall proceed to offer for your consideration a few remarks on odontalgia, its treatment, &c. The subject having been left discretionary with myself, I have been influenced in its selection more on account of its practical importance and a desire to benefit the junior members of the profession, by inviting attention to doctrines and principles with which it is connected, than from any expectation of being able to present any new doctrine or theory to the elder or more experienced members of this association.

There is no disease, in the treatment of which, more importance is attached to a judgment capable of discriminating correctly as to the pathological condition of parts, the curative indications and the necessity and practicability of fulfilling them, than in tooth-ache. Nor is there any disease to which the human body is subject, of more frequent occurrence, or that cannot be borne with more patience, and for which the means of relief are sought with greater avidity, nor comparatively few in which so rapid and extensive an influence is exerted in the production of local and general disturbance, and where there is so much difficulty in deciding upon the pathological condition of the structures involved in the train of morbid phenomena, than the one now under consideration.

Every aggravated and protracted case of tooth-ache, therefore, may be regarded as being productive of more or less mischief, according to the circumstances under which it occurs. Even under the most favorable, the evils resulting from it are often more to be feared than the loss of a sound tooth, especially where recourse is had to many of the therapeutic agents which have been recommended for the cure of this affection. The local and constitutional effects produced by these agents are sometimes more calamitous than the disease which they are intended to cure.

In defining the varieties of tooth-ache, I shall make no attempt at innovation. I shall therefore only consider two kinds, namely, idiopathic and sympathetic. The former resulting from or dependent on local irritation and inflammation of the lining membrane, and of the peri-dental membranes when the vitality of this has been previously destroyed; and the latter from constitutional causes or a transfer of nervous irritation.

The first is by far the most prevalent; the other seldom or never occurring without being invited by some local, physical, or morbid predisposition, and in either case the frequency and violence of the malady is materially augmented or modified by the presence or absence of reciprocal morbid associations.

An experience of nearly twenty-five years, exclusively devoted to the various duties of the dental profession, has not been suffered to pass without endeavoring to discover some plan of treatment, or remedy, other than that afforded by the forceps, which might prove a safe and radical cure of idiopathic odontalgia, and thus secure the restoration of the diseased organ. But, in comparatively few instances have I succeeded either with the treatment which I have been able to suggest, or that recommended by others. Subsequent morbid developments have sooner or later, rendered extraction necessary.

I do not wish it to be inferred, however, from what I have said, that this form of tooth-ache, cannot, under any circumstances, be subdued, but the number of cases permanently cured, are comparatively so few, that the principle laid down by medical writers, should apply here, that where any particular treatment of a disease more frequently fails than succeeds, it should be abandoned. There is no precedent more worthy of adoption than this, and in the treatment of no class of diseases, will it be found more applicable, than in the one now under consideration. And if admonition and precept be not amply sufficient to establish the correctness of this principle, observation and experience will not fail to do so.

The proper treatment, therefore, in the majority of cases, for this description of tooth-ache, is the removal of the diseased organ, and it is greatly to be regretted that the dread of anticipated pain should, as frequently as it does, prevent persons laboring under this form of the malady, from submitting to the operation, and that so many dentists substitute other treatment, which at least, is inefficient, and subjects the unfortunate sufferer to a train of evils far greater than mere tooth-ache itself. Such practice cannot be regarded in any other light than as empirical. The surgeon dentist, should always under such circumstances, endeavor to persuade his patient to submit to the removal of the tooth, by pointing out the bad effects that will inevitably result from its retention in the mouth. He should never sacrifice principle by the adoption of a temporizing treatment, for the mere gratification of the feelings or whims of his patient, when it is possible to do otherwise. But the fault is not always with him. His reasonings sometimes fail to overcome the timidity of his patient, and when this is the case, he is often compelled to yield to the force of circumstances.

The necessity for promptitude and decision in the treatment of this disease, becomes apparent when we consider the physical peculiarity of organization of the teeth, and the relation they sustain to each other and to the circumjacent and even remote parts of the body. Although when free from all constitutional predisposition or bias to disease, they are capable of resisting, for a long time, the influence of local and other causes, yet, when attacked by caries or almost any other affection to which they are liable, they are never restored by the sanative powers of nature. In this respect they differ from other organs of the body, and hence their treatment is rendered more important and difficult. Their restoration, therefore, must depend upon the skill and judgment of the practitioner.

But although the dentist derives no aid from the recuperative powers of the economy in the treatment of the diseases of the teeth themselves, yet a healthy condition of the general system, and more especially of the parts contiguous to these organs, is of the utmost importance to the attainment of the end for which his treatment is instituted.

Of all the obstacles encountered in the treatment of disease, and the most difficult to be overcome, are those which result from physical causes, and in the treatment of no class of which, are they so frequently met with, as in those of the teeth. Owing to their peculiarity of organization, operations upon them, with few exceptions, will not admit of mediocrity in their performance. Before deciding upon the course of procedure proper to be adopted in the treatment of diseased teeth, the dentist when called upon for his services, should first direct his attention to the state of his patient's general health, the physical characteristics and peculiarities of his teeth, the nature and extent of the disease, and the causes which have produced it, bearing in mind that his success will depend as much on a thorough knowledge of all the circumstances attending the case, as upon the neatness and skill of his operation.

It should also be recollected that tooth-ache, usually treated of as a disease, is only a symptom or the effect of disease, and is always preceded either by local physical predisposition, excited by constitutional disturbance, as in the sympathetic form of the affection, or by inflammation of the peri-dental membrane, resulting from mechanical violence or the irritation caused by dead teeth, or from irritation and inflammation of the lining membrane, produced either by mechanical injury or the presence of acrid substances, as in the idiopathic form of the malady.

For the last named form of the disease, any treatment but extraction, is only productive of temporary relief, while the disease, of which the pain is only the effect or symptom, takes its course, oftentimes giving rise to other local as well as constitutional affections, which sometimes assume a very aggravated form. If the experience of the most scientific and skilful practitioners that have ever lived, could be ascertained, I think it would be found, that in the adoption of other treatment than this, they have often been disappointed in their expectations, and that while they and their patients have been lulled into imaginary security, by the temporary relief of the latter from pain, the malady itself has continued and become the cause of other, and sometimes more formidable diseases—furnishing the most convincing proof of the errors that had been committed in the previous treatment.

As unpardonable as it may be to sacrifice a sound tooth, or one that might be restored to health, through mistake, ignorance or otherwise, and great as the loss certainly is, of such a tooth, it sinks into insignificance, when compared with the consequences resulting from the retention of a tooth in the mouth that cannot be restored to health, or suffered to remain without exerting a morbid influence upon the surrounding parts and nervous system generally.

If it were necessary, hundreds of well authenticated cases might be adduced in proof of the fact, that it is not necessary for a tooth to cause pain, or give rise to alveolar abscess, to be productive of even the most serious consequences. The records of medicine furnish numerous examples of this sort, and cases are daily met with, where, after every remedy had been tried that could be suggested by the skill and ingenuity of man, without success, cures have been speedily and permanently effected by the removal of diseased teeth, and even where they have not been productive of pain or suspected as the cause of the mischief. Many such have fallen under my own observation.

With facts such as these before us, it is not surprising that the younger and more inexperienced members of the profession, should often be deceived and encouraged to persist in the repetition of a practice that has done more harm than could possibly have been produced, had no other remedy than extraction ever been resorted to.

When I see an advertisement setting forth the cure of tooth-ache, indiscriminately, without reference to its cause, or the circumstances connected with it, by secret nostrums, rendering extraction unnecessary, I take it for granted that the author is either a quack or a knave, and most probably both.

As a general rule, in the treatment of sympathetic tooth-ache, extraction is not necessary. But exceptions are sometimes met with. When the proper treatment has been neglected, until inflammation and suppuration supervene, involving the peridental membrane, the extraction of the aching tooth becomes necessary. From the many cases of this description, which have fallen under my own immediate observation, I will mention one.

In April, 1830, I visited, by request, a Mr. W——, a respectable merchant of Hopkinsville, Ky., aged between forty-five and fifty. His general health for the preceding two years had been greatly impaired, but up to which time, from infancy, it had been good, had never lost but three teeth, and these from the effects of dry, black caries. At the time when I first saw him, all his teeth were sound except the sides of two, which had originally been in contact with two that had previously been extracted, and the caries of these had not penetrated sufficiently deep to require the operation of filling.

The tooth of which he complained was the right superior cuspidatus; it was not in the least affected with caries, nor had it been injured by mechanical violence, but it had been more or less painful for eight or ten days, was very tender and sensitive to the touch, and the gums around it, were very much inflamed and tumefied.

After a thorough investigation of the case, I decided on the extraction of the tooth, and having performed the operation, I split it open lengthwise, and as I had suspected, suppuration of the pulp and lining membrane had taken place.

The patient, although greatly relieved by the operation, suffered more or less pain for several days, but was gradually restored to health.

It may not be amiss to observe, that some have been in the habit, in cases of this sort, to adopt the practice recommended by Mr. Fox; namely, to trephine the tooth, or rather to drill a hole through it into the internal cavity, for the escape of the matter, and after the pain and inflammation has subsided, to cleanse and fill it with gold—proposing thereby to restore the organ to health and usefulness. It is scarcely necessary to say that this practice is in direct opposition to all surgical experience, and has rarely, if ever, been attended with success.

Cases are continually occurring, in which the young and inexperienced practitioner, finds it difficult to determine on the practice most proper to be pursued, but it is hardly necessary to observe, that he should never extract a tooth, however strongly he may be urged to do so, unless, upon careful examination, he finds this to be the only correct curative indication. It is a well established pathological fact, that parts primarily affected, do not always complain for themselves; the pain is often felt in a remote locality. Hence, sound teeth are often referred to as the seat of the disease, when in fact it is seated in some tooth quite remote from it, and are frequently sacrificed both by medical and dental practitioners. But the thoroughly educated and experienced dentist, will always be able to detect the organ that has produced the disturbance, even though it be remote from the one in which the pain is felt.

As a general rule, palliative treatment in idiopathic tooth-ache, is not advisable, except in the deciduous teeth, and then, only when inflammation and suppuration has not destroyed their vitality and rendered them sources of irritation to the surrounding parts. In this case, the morbid effects they exert upon the adjacent and contiguous parts, as well as upon the permanent teeth, which are at this time being formed, is productive of more injury than could be produced by their removal.

All teeth, whether belonging to the first or second dentition, which have given rise to alveolar abscess, should be immediately extracted, except in some very special cases, where their retention is called for by some very pressing necessity; and even here, the peculiar circumstances of the case which may require their presence only, can justify the exception to the rule. But important as is the retention of the temporary teeth, until the period when they should be removed by the operations of the economy to give place to more substantial successors, a deciduous tooth, after having become a source of morbid irritation, should not be permitted to remain in the mouth, and under such circumstances, the dentist should not hesitate to remove it. Moreover, the premature loss of the temporary does not always, and of necessity prevent a natural and regular arrangement of the permanent teeth. I might adduce many examples, if it were necessary, in proof of the correctness of the assertion, yet as their premature loss is exceedingly liable to do so, the practice as I have before stated, should only be adopted as a dernier resort, as the least of two evils.

A temporary tooth seldom aches, until it has been attacked by caries, and then, not until the disease has penetrated to the nerve cavity. It is therefore to be regretted that it is so frequently permitted to attack them, when by timely and proper attention, it might, in the majority of cases be prevented, until the fulfilment of the indications of nature. Sometimes, however, owing both to defective physical organization and morbid constitutional influences, the best directed efforts to ward it off, will be defeated. In this case, its progress should be arrested before it has penetrated to the cavity of the tooth.

I am aware that the resources of art are seldom resorted to, for arresting the progress of caries in the teeth of first dentition, but I feel warranted, from experience, in recommending their application. Disease may, generally, be as successfully treated in these teeth, as in those of second dentition.

For the last few years, arsenic, has been extensively employed as a remedy for tooth-ache, and when the disease is dependent on inflammation of the lining membrane, it has proved successful, but inasmuch as it destroys the vitality of the tooth, the organ is rendered obnoxious to the surrounding parts. In consequence of which, in nine cases out of every ten, where the cavity in the tooth is afterwards filled, it gives rise to alveolar abscess. The practice, therefore, is unscientific, and productive of decided injury. For allaying simply sensibility of the bony structure of a tooth, it may sometimes be advantageously employed. But in using it even for this purpose, much judgment and care are necessary. If it be placed in the cavity of a tooth of a very young person, and which is of a soft structure, it will oftentimes destroy the pulp, and cause an effusion of blood in the internal cavity, imparting to the organ, at first, a pale pink color, then that of a dark brown or purple, and ultimately give rise to alveolar abscess. No one article has been more extensively and generally employed in the treatment of tooth-ache, during the last five or six years than arsenic, and for some time it promised to be an invaluable remedy, but the pernicious effects which have resulted from it, should forever preclude its use as an agent for the destruction of the lining membrane of a tooth.

In thus giving expression to my strong disapprobation of the use of this article, I am happy to believe, that it is already discarded from practice by most of the better informed and more scientific members of the profession. There may be some and perhaps many respectable practitioners who, not having had an opportunity of observing the ultimate effects which result from it, still use it, but at present, in this country, I believe there are few such, and that its employment is chiefly confined to that class who advertise secret nostrums for the cure of this most painful and aggravating affection. In most, if not in all of the nostrums of the day, set forth and advertised as infallible remedies for tooth ache, this constitutes the active ingredient, and is the essential agent upon which their authors rely for their temporary success.

It is unnecessary to say, that of all the other therapeutic agents that have been suggested and applied, for the cure of idiopathic tooth-ache, none have proved infallible. Many have been found to give temporary relief, to assuage the pain for a short period, for a few hours, or perhaps days; after which, it has recurred, in most instances, and often with increased violence. When, however, it is dependent upon the presence of some local irritant, the removal of this will often afford temporary relief, but a permanent cure will seldom be effected, except by the removal of the tooth.

But tooth-ache, resulting from a transfer of nervous irritation, or general constitutional disturbance, can often be speedily and radically cured. But in the treatment of this description of the disease, the remedies should accord with the peculiar indications of each individual case. An emetic or cathartic will sometimes give immediate relief. Pregnant females are peculiarly subject to this form or variety of tooth-ache, and in cases of this sort, a Dover's powder and pediluvium on going to bed, will often be highly advantageous. I have known it in many instances to give speedy relief.

But to enter into a detailed description of the curative indications of sympathetic tooth-ache in every case which is liable to occur, would be to extend my remarks to too great a length, I shall therefore conclude, by simply observing, that the remedies must, in most cases, be addressed to the general system; or to organs remote from the teeth, and can only be determined by the pathological and therapeutical knowledge of the practitioner. To attempt to cure this form of the disease by local treatment, would only be to protract the sufferings of the patient, and in the end, to realize nothing but disappointment.

